# *Lariophagus distinguendus* (Hymenoptera: Pteromalidae) (Förster)—Past, Present, and Future: The History of a Biological Control Method Using *L. distinguendus* against Different Storage Pests

**DOI:** 10.3390/insects7030039

**Published:** 2016-08-01

**Authors:** Steffi Niedermayer, Marie Pollmann, Johannes L. M. Steidle

**Affiliations:** Institute of Zoology/Animal Ecology 220c, Hohenheim University, Garbenstr. 30, Stuttgart 70599, Germany; steffini@uni-hohenheim.de (S.N.); marie.pollmann@uni-hohenheim.de (M.P.)

**Keywords:** biological pest control, Pteromalidae, *Lariophagus distinguendus*, *Sitophilus* sp., *Stegobium paniceum*, stored product protection

## Abstract

Legal requirements and consumer demands for residue-free products pose a big challenge for pest control in grain stores. One possible alternative to chemical insecticides is biological pest control with the pteromalid wasp *Lariophagus distinguendus* against the weevils *Sitophilus granarius*, *S. oryzae* (Coleoptera: Dryophtoridae), and many other storage pest beetles. The use of this wasp as a biocontrol agent was already suggested in 1919 by Prof. Dr. Hase [1]. Despite many studies on host-finding and behavioral biology, the applied aspect was neglected until 1994. Nowadays the wasps are commercially available and can now even be reared on-site, facilitating their use tremendously. This review highlights the milestones in *L. distinguendus* research, gives insights in current studies, and ventures a glimpse into the future.

## 1. Introduction

Stored grain is threatened by a vast number of storage pests, mostly insects [2]. Nowadays integrated pest management strategies (IPM) have superseded the excessive application of chemical insecticides used against storage pests in the past. An important element of IPM is biological pest control, a technique that uses living organisms, so-called natural enemies, to suppress the populations of certain harmful animals or plants below the economic injury level [3]. To protect stored products from storage pests, antagonists that naturally occur in storage and which are already adapted to the storage environment [4,5] can be mass-reared (augmentative control) and released. The storage environment is especially suitable for biological pest control [6,7]. Temperature and humidity conditions are more stable than in the field. Furthermore, a storage building is a limited confined space reducing the risk of antagonists migrating from the application site [7]. One of the main storage pests in temperate climates in Europe is the granary weevil *S. granarius* [8]. It can be attacked by the parasitoid wasp *L. distinguendus*, which is a solitary ectoparasitoid with a wide host spectrum. It attacks different developmental stages of at least 17 beetle species in six families. However, four of the 17 species are described as hosts only once [9,10]. The beetles attacked are mostly storage pests belonging to the families Dryophtoridae, Chrysomelidae, Anobiidae, Bostrychidae, Curculionidae, and Ptinidae [9]. Only hosts which develop concealed in seeds, grain kernels, or cocoons are attacked [11,12]. The use of *L. distinguendus* as a biocontrol agent was already suggested in the early 20th century. Since then, many studies have dealt with different aspects of its biology. This review will summarize earlier and present work and will venture a glimpse on the future role of the wasp in biological pest control.

## 2. Biology of *Lariophagus distinguendus*

*L. distinguendus* is a solitary ectoparasitic idiobiont. It is specialized on late larval, pre-pupal, and pupal host stages [13,14,15]. After encountering an infested seed or cocoon, female wasps show a distinct sequence of behaviors (drumming, tapping, and drilling) by which they determine the presence and suitability of a host [13]. If an appropriate host is detected, the female paralyzes the host and thereby stops its development [14,16]. Afterwards the female lays one egg on each host. During development the wasp larva feeds on the host larva, molts, pupates, and then hatches. The developmental time and the number of offspring strongly depend on the surrounding temperature [17,18], but showing protandry, males always hatch earlier than females [19]. Mating typically takes place right after the females hatch, at the hatching site.

## 3. The 1920s and 1930s

Early work on *L. distinguendus* mostly focused on the morphology of the wasp [1,20], its systematics and synonymy [1,20,21], and its general biology, e.g., mating [1], oviposition [13,22], and cleaning [23]. Besides the general interest in the biology of the wasps, the applied aspect has already been considered as well. However, research was descriptive rather than experimental. Burkhardt, working at the Department of Plant Diseases at the Kaiser Wilhelm-Institute for Agriculture in Bromberg, Germany, stated that a closer look at the impacts of the wasps on the host’s life is advisable [17]. Hase, chief of the Department of Agricultural Zoology of the Federal Biological Institute in Berlin, Germany, mentioned the need for a more thorough understanding of the wasp since it is a parasitoid of the “dreaded black weevil *Calandra granaria* L. (=*Sitophilus granarius*)” (Dryophtoridae) [1]. In the *Reports of the Grain Pest (War) Committee* Goodrich examined grain infested by different Coleoptera [24]. He revealed that *Calandra oryzae* (=*Sitophilus oryzae*) (Dryophtoridae), *C. granaria* and *Rhizopertha dominica* (Bostrichidae) were parasitized by *L. distinguendus*. He also stated that “since the Hymenoptera are sometimes very numerous they must seriously interfere with the multiplication of the beetles, and must therefore be reckoned as a useful factor in moderating their ravages.” The idea of using *L. distinguendus* as a biological control agent against *S. granarius* was born. In subsequent publications Hase describes the “host-shift” of *L. distinguendus*, specifying another storage pest, the drugstore beetle *Stegobium paniceum* (Anobiidae), as a host [13]. In 1926, Ryabov looked at “the possibilities of applying the parasitic method of control in the case of granary pests” and strongly suggested the use of *L. distinguendus*, especially in storages where no other control methods could be applied [14]. About 10 years later, Smirnov and Polejaeff conducted the first experiments dealing with the wasps’ ability to detect infested grain among uninfested grain [25]. They showed that *L. distinguendus* possesses an astonishing host-finding ability, being able to find only a few infested grains within 230,000 uninfested ones (1937).

## 4. The 1950s and 1960s

It was more than 15 years later that Kashef resumed the studies on *L. distinguendus.* He noted that a thorough knowledge about the biology of *L. distinguendus* and the biology of its hosts is necessary for a successful application of the parasitoids against storage pests [15]. Therefore, he studied the morphology and biology of *L. distinguendus* and several of its hosts [15,26,27,28,29,30]. He concentrated on the determination of the host stage most suitable for parasitization and the wasps’ fecundity on different hosts [15,29,30]. He compared the reaction of *L. distinguendus* to known hosts such as *S. paniceum* and *S. oryzae* with beetles which had not been described as hosts before, such as the lesser grain borer *R. dominica*, the Australian spider beetle *Ptinus tectus,* and the smooth spider beetle *Gibbium psylloides* (Ptinidae) [30]. In this context he also brought up the question of “the natural host” and the idea of a separation into biological races due to a particular host preference and subsequent ecological isolation. Another focus of Kashef’s work was on cues guiding the wasps to their hosts [31]. He was able to disprove the idea of sound being a host-finding cue as stated by Smirnov and Polejaeff [25,29]. Instead he showed that *L. distinguendus* is attracted by the odor of its different hosts. Even though Kashef’s main focus was not on stored products protection, his work was the basis of many applied (and fundamental) studies that were yet to come.

## 5. The 1970 and 1980s

In the 1970s and 1980s, the first paper with a focus on an applied research approach was published by Gonen and Kugler, who looked at the biology of *L. distinguendus* on *S. oryzae* with the goal “… to obtain more information […] of this wasp as a parasite of the rice weevil, especially those aspects which might influence its efficiency as a natural control agent” [19]. However, based on their sex ratio and longevity experiments, Gonen and Kugler doubted that *L. distinguendus* would be able to “cope with the rate of increase of the host population”. They stated that the high number of male offspring in early larval host stages and the short lifespan of adult females were the main factors making this method ineffective. However, wasps used by Gonen and Kugler could choose between different host sizes, therefore producing sons on smaller hosts and daughters on larger ones. Putters and van den Assem could show that the absolute size of the host is not important in sex-regulation [32]. Wasps will always define a host as small or large compared to the other hosts available; therefore, in an environment of predominantly small hosts, *L. distinguendus* would still produce male and female offspring [32]. Thus, the objections of Gonen and Kugler could be disposed of. Several other studies were conducted by van den Assem [33,34] and his colleagues Bellows, Charnov, and Werren [35,36,37] and *L. distinguendus* was established as a model organism for general behavioral, evolutionary, and population ecology. The main focus of their work was on sex-ratio regulation and population dynamics [35,36,37]. They showed that parasitoids, with diploid females and haploid males, are able to adjust the sex ratio of their offspring to certain ecological conditions such as host size [38]. As part of their studies, two more hosts of *L. distinguendus*, the bean weevils *Callosobruchus chinensis* and *C. maculatus* (Chrysomelidae), were specified [39].

## 6. The Early 1990s

In the early 1990s, a working group from Korea started working with *L. distinguendus*. Their research was application-oriented, focusing on the ability of *L. distinguendus* to suppress populations of the rice weevil *S. oryzae*. First Yoo et al. concentrated on different developmental stages of *S. oryzae* and their suitability for *L. distinguendus* [40]. Later, a special focus of their work was on the impact of abiotic factors, namely temperature, on the functional and numerical response to different host densities and the development of the wasps [18,41]. Ryoo and his colleagues were able to rebut objections of Gonen and Kugler from the 1970s regarding the short life of adult females or the high number of cases in which two eggs per host were laid [19]. They could show that the lifespan of female wasps was long enough for a sufficient parasitization of hosts and that two eggs per host normally only occur rarely or at low host densities when the waste of eggs is unimportant [18,33,42]. Ryoo further stated that “the biological characteristics of *L. distinguendus* may be compatible with efficient control of rice weevil populations” [18]. However, Hong noted that the regulation of rice weevils “seems unlikely to occur under lower temperature conditions” [41]. A later study looked at the impact of a second parasitoid, *Anisopteromalus calandrae* (Hymenoptera: Pteromalidae), when applied simultaneously with *L. distinguendus*. Even though *A. calandrae* is considered to be dominant, the two wasps coexisted until the host population was suppressed [43].

## 7. From the Late 1990s–Present

Starting in the mid-1990s, the increased demand for pesticide-free products and environmentally safe pest-control methods intensified the studies on *L. distinguendus* tremendously. In the late 1990s and early 2000s, the main center of research was in Berlin/Germany at the BBA (Biological Federal Agency, Berlin, Germany) and the Applied Zoology Department at the Freie Universität Berlin. At that time one major point of interest was the identification of host searching cues used by *L. distinguendus* to detect suitable hosts (*S. granarius)* [44]. Based on the first descriptions by Thorne and Jones concerning the importance of learning in searching behavior [45], the influence of associative learning was also investigated [44,46,47]. Cues used during the different steps of host searching were investigated for different host complexes, revealing that for host finding as well as for host recognition semiochemicals present in the host’s feces are used [48,49,50,51,52,53,54]. Chemical studies showed that the substances in the host’s feces (*S. granarius* on wheat) which trigger (at least) host recognition are compounds commonly found in grain (e.g., tocopherol) and compounds from the host (cholesterol) [54]. These findings also explain why wasps not only respond to grains infested with *S. granarius* larvae but also to healthy wheat grains, a reaction probably due to associative learning. HIS (herbivore-induced synomones, either actively produced or released as a passive physiological reaction by attacked seeds to the feeding activity of the host) were also identified to play a role in host localization [55]. Furthermore, Ruther and Steidle found that *L. distinguendus* also reacts to volatiles emitted by astigmatic mites, again a cue most likely learned associatively [56]. In the context of host recognition, Ambriz et al. looked at cues used to locate *Lasioderma serricorne* (Coleoptera: Anobiidae), another host of *L. distinguendus* [57,58]. They found that extracts from host cocoons trigger host-recognition behavior and oviposition in *L. distinguendus* and that experience has an enhancing influence on oviposition. However, semiochemical cues are not only used for host (habitat) location but also to avoid unsuitable host habitats, e.g., habitats infested by mold [59].

Besides these basic studies, another major point of interest was the applied use of *L. distinguendus*. Schöller together with his colleague Prozell established the company Biologische Beratung Ltd, which was the first to produce and sell *L. distinguendus* commercially. Steidle and Schöller conducted the experiments on the dispersal ability of the wasps [60]. They showed that *L. distinguendus* is able to locate hosts up to 4 m vertically and horizontally in bulk grain. Steidle and Reinhard showed that wasps released in grain storage will be arrested by grain odors and low humidity and will remain in the storage environment [61]. Within the project “Strategies for the Regulation of Storage Pests in Storages and Factories for Organic Products”, further studies concerning the application of *L. distinguendus* were conducted [62]. By means of studies and an intensive literature research, live parameters of the parasitoid were collected and a decision support application software for farmers and operators was developed [62]. Furthermore, a release schedule was calculated [62,63,64,65]. After the appointment of Steidle as the head of the Department of Animal Ecology at Hohenheim University, Germany, another center of *L. distinguendus* research was established. Steidle focused on basic research about the mechanisms of speciation with a focus on early learning and courtship behavior, using *L. distinguendus* as a model system [66]. It could be shown that the species *L. distinguendus* de facto consists of two species. While one of these two species is a generalist on many different hosts, the other apparently has a narrower host range and is unwilling to parasitize *S. granarius*, a fact that could have a huge impact on the application [66]. Furthermore, Steidle picked up earlier applied work looking at the dispersal ability of *L. distinguendus*, this time studying the dispersal of the wasps outside the commodity, applied as an empty storage treatment against residual pest populations [67]. Results indicate that host location outside the commodity is strongly influenced by factors such as light and that the number of wasps released should be higher than the number recommended for use inside the commodity. Additional research on dispersal and host finding was conducted by Adarkwah et al. who looked at the ability of *L. distinguendus* to reach hosts inside jute bags and bulk maize [68,69]. They showed that the wasps, depending on mesh size, are able to penetrate the bags and reduce pest infestations by 81%, while reduction was about 37% one meter inside the maize bulk. Further applied research was done by Hansen et al. who first focused on the influence of low temperature on the development and winter survival of *L. distinguendus* and its host *S. granarius*, showing that the wasps are able to develop, emerge, and produce viable offspring after being exposed to 6 °C for 15 weeks [70,71]. Since reproduction values of the wasps at low temperatures are higher than those of the weevils, *L. distinguendus* should be able to control granary pest populations even at low temperatures. In this context, Niedermayer et al. examined the parasitization ability of *L. distinguendus* compared to that of the closely related parasitoid *A. calandrae* under extreme temperature conditions [72]. *L. distinguendus* performs better at lower temperatures whereas *A. calandrae* performs better at warmer temperatures. Therefore, a combined application has been suggested.

Further studies concentrated on special circumstances possibly affecting the efficiency of *L. distinguendus*. Belda and Riudavets showed that in cases where the two pest species *S. oryzae* and *R. dominica* co-occur in the same storage, only populations of *S. oryzae* but not populations of *R. dominica* are affected by *L. distinguendus* [73]. Lucas and Riudavets investigated the combined use of *L. distinguendus* with other pest control methods such as mechanical polishing treatment of rice (kernels are rubbed and lose their pericarp layer), showing that even though the reduction of pests was enhanced, mechanical treatment also had a lethal effect on the wasps [74]. Additional studies regarding the combination of parasitoids with other control methods were conducted by Hansen. She investigated the use of entomopathogenic fungi and *Bacillus thuringiensis* in maize and could show that both treatments had negative effects on the efficacy of the wasps [75,76]. Schöller and Prozell looked at a combined use of diatomaceous earth and *L. distinguendus.* No positive effect of the combination could be shown [77].

To facilitate the application of *L. distinguendus* Steidle and Niedermayer developed a mass rearing device [9]. The device contains a self-sustaining system of host medium, hosts, and parasitoids and continuously releases wasps directly on site in storages over several months. Together with Schöller and Prozell from Berlin, the use of parasitoids in long-term storages is currently examined.

## 8. Status Quo and Outlook

Research has come a long way since the first observations by Hase, Ryabov and Kashef. At present *L. distinguendus* is produced commercially and distributed by at least 11 companies in central Europe (Internet inquiry using Google). Wasps are reared in the laboratory, shipped in units of about 40 wasps in plastic tubes, and are then released on location [78]. In storages, two to three releases per year are recommended (inundative release); for application against Ptinidae in buildings, wasps should be released monthly. The key market concentrates on rather small farms, mills and retail stores, most of which are in the organic sector. For the future an enhanced involvement in IPM in conventional farms and companies is desirable. Furthermore, the applicability of *L. distinguendus* in large storage facilities with long-term storage is currently being examined. To facilitate the application of the wasps, the rearing box of Steidle and Niedermayer should be completed and commercialized.

Future research should focus on the selection of strains most suitable for the different pest species as it is commonly done, e.g., for *Trichogramma* sp. The selection of strains should also play a role depending on the different climate conditions at the application sites.

Additionally, more research is needed concerning the application of *L. distinguendus* against the nuisance *G. psylloides* in households. Even though *G. psylloides* has been described as a host of *L. distinguendus*, parasitization was only observed under the artificial conditions of laboratory experiments. It is yet unclear how the wasps manage to reach their hosts which normally hide behind walls and in the ceiling. Currently it is recommended to drill holes in walls and ceilings to facilitate accessibility of the hosts. However, nothing is known about the distance between release points or a possibly negative influence of abiotic factors such as light or temperature gradients. To enhance the host-finding success and render the application of *L. distinguendus* as effective as possible, more research is necessary.

Summing up the results from recent research on the use of parasitoids for the control of stored products pests, we recommend the following guidelines:

(1) When releasing wasps, their host-finding ability must be considered. Release points should be within 4 m of potential pest sites in bulk grain [60]. For application against residual pest populations in empty storage, release points should be, at most, 10 m apart [67] and cover no more than 10 m^2^ [78]. The host-finding ability of hosts in bags strongly depends on the bag material. Wider mesh sizes facilitate the penetration ability of *L. distinguendus* [68].

(2) When *L. distinguendus* is applied outside the commodity, a significantly higher number of wasps should be applied compared to recommendations for the application in the grain bulk, because a certain amount of wasps does not manage to reach hosts [67,68,77]. Distractions due to light and obstacles must be considered and eliminated if necessary [67].

(3) To balance different host-finding ranges, a mixture of *L. distinguendus* and the closely related parasitoid *A. calandrae* should be applied [67].

(4) *L. distinguendus* strains should be selected in accordance to the pest species against which they are applied [66]. Especially strains originating from the host *S. paniceum* might be specialized on that host and are thus inefficient against other hosts [66].

(5) Temperature should always be considered when releasing parasitoids, especially in empty storage treatments. *L. distinguendus* should be released during the cold season or in colder geographic regions. During summer or in warmer areas *A. calandrae* is a suitable alternative [70,71,72].

(6) The combined application of *L. distinguendus* and other control methods, such as mechanical polishing, and the use of entomopathogenic fungi or diatomaceous earth are not recommended [74,75,76,77].

(7) When using *L. distinguendus* against pests in storage, wasps should be released four weeks after harvest and typically again in fall and spring [77]. To facilitate the application of the wasps, the use of a mass rearing device is recommended. After the initial set-up the device releases wasps continuously over several months [9].
